# Multiple TORC1-Associated Proteins Regulate Nitrogen Starvation-Dependent Cellular Differentiation in *Saccharomyces cerevisiae*

**DOI:** 10.1371/journal.pone.0026081

**Published:** 2011-10-17

**Authors:** Sunil Laxman, Benjamin P. Tu

**Affiliations:** Department of Biochemistry, University of Texas Southwestern Medical Center, Dallas, Texas, United States of America; Institute of Developmental Biology and Cancer Research, France

## Abstract

**Background:**

The budding yeast *Saccharomyces cerevisiae* undergoes differentiation into filamentous-like forms and invades the growth medium as a foraging response to nutrient and environmental stresses. These developmental responses are under the downstream control of effectors regulated by the cAMP/PKA and MAPK pathways. However, the upstream sensors and signals that induce filamentous growth through these signaling pathways are not fully understood. Herein, through a biochemical purification of the yeast TORC1 (Target of Rapamycin Complex 1), we identify several proteins implicated in yeast filamentous growth that directly associate with the TORC1 and investigate their roles in nitrogen starvation-dependent or independent differentiation in yeast.

**Methodology:**

We isolated the endogenous TORC1 by purifying tagged, endogenous Kog1p, and identified associated proteins by mass spectrometry. We established invasive and pseudohyphal growth conditions in two *S. cerevisiae* genetic backgrounds (Σ1278b and CEN.PK). Using wild type and mutant strains from these genetic backgrounds, we investigated the roles of TORC1 and associated proteins in nitrogen starvation-dependent diploid pseudohyphal growth as well as nitrogen starvation-independent haploid invasive growth.

**Conclusions:**

We show that several proteins identified as associated with the TORC1 are important for nitrogen starvation-dependent diploid pseudohyphal growth. In contrast, invasive growth due to other nutritional stresses was generally not affected in mutant strains of these TORC1-associated proteins. Our studies suggest a role for TORC1 in yeast differentiation upon nitrogen starvation. Our studies also suggest the CEN.PK strain background of *S. cerevisiae* may be particularly useful for investigations of nitrogen starvation-induced diploid pseudohyphal growth.

## Introduction

In response to a variety of nutrient and environmental conditions, many fungi undergo developmental switches between unicellular yeast and filament-like forms [1], [2]. When severely deprived of nitrogen, diploid yeast cells can initiate pseudohyphal differentiation, where cells elongate and adopt a unipolar budding pattern, spreading across the growth medium [3]. In haploid cells, phenotypically similar cellular differentiation is observed in response to various nutrients and stresses such as growth on non-fermentable carbon sources, variable glucose levels or the presence of alcohols [3], [4], [5], [6], [7], [8]. Under such conditions, cells invade the growth medium and form filament-like clusters (termed filamentous growth in the remainder of this manuscript, and not to be confused with other filamentous fungi). These responses collectively appear to be foraging mechanisms through which yeast overcome a variety of nutritional and environmental stresses [9].

These various types of filamentous growth appear to be under the final control of the cAMP/PKA and Snf1 pathways [7], [10], [11], [12], [13], [14], [15] as well as the MAPK pathway [5], [6]. The Snf1 pathway in particular may be central to these growth responses since it responds to both glucose and nitrogen limitation, and also regulates a host of genes whose induction are required for filamentous development particularly under nitrogen limitation [11], [16], [17]. More recent work describes a quorum signaling pathway by aromatic alcohols that controls filamentous growth [18], [19]. These alcohols are regulated by nitrogen levels and stimulate morphogenesis through the expression of the cell surface flocculin *FLO11*, which is required for cell aggregation and invasion. Yet, the differences between these various types of filamentous growth behaviors, particularly with respect to the systems that initially sense these diverse inputs, are not fully understood. Despite the known roles of the PKA, Snf1 and MAPK pathways as the effectors of these developmental changes, it remains unclear if all of these diverse stress and nutrient inputs are received by the same pathways, or if the different inputs are actually sensed by different pathways which in turn orchestrate different responses [9]. Thus, the extent of similarity between the developmental responses to either carbon or nitrogen restriction remains an open question.

The TORC1 is a highly conserved and essential protein complex in eukaryotes that senses and responds to numerous signals, particularly nitrogen but also glucose, oxygen levels and environmental stress [9], [20]. TORC1 subsequently controls cell growth in part through the activation of ribosome biogenesis and the translational machinery [9], [20], [21]. TORC1 consists of multiple protein subunits that can associate dynamically, and the full range of interacting partners remains unclear. The core, conserved components of TORC1 include the Tor kinase (Tor1p and Tor2p in yeast), Kog1p (the yeast ortholog of RAPTOR), Lst8p and Tco89p [22], [23]. The Tor kinase activity is inhibited allosterically by the natural product rapamycin, in complex with the prolyl isomerase FKBP12 [24], [25]. The major effectors of downstream responses of TORC1 are the nutrient-responsive kinase Sch9p [26], [27], and the protein phosphatase 2A (and PP2A-like phosphatases) in conjunction with Tap42p [28], [29], [30], which collectively orchestrate a host of responses to various environmental and nutrient inputs through TORC1. The TORC1 pathway has now been well-established as a key nutrient and metabolic sensor that regulates the growth of eukaryotic cells. Yet, the various factors that sense nutrients, particularly amino acids, and subsequently activate TORC1 remain elusive.

Our initial efforts were directed towards a robust biochemical purification of the TORC1 from yeast grown under different nutrient conditions. From these purifications, we identified several proteins that directly associated with TORC1. Mutants of a subset of these identified proteins are listed within the yeast genome to display filamentous growth phenotypes. Since the relative role or importance of TORC1 in yeast filamentous growth is still fairly unclear, in this study we specifically focused only on this subset of identified proteins. Using prototrophic yeast strains from two different genetic backgrounds (CEN.PK or Σ1278b), we present data suggesting that nitrogen starvation-dependent diploid pseudohyphal growth may be regulated by the TORC1. This may represent one means through which budding yeast distinguishes between the numerous diverse inputs that induce filamentous differentiation upstream of the PKA, Snf1 and MAPK pathways. Our work also suggests the CEN.PK strain background will be useful for the exclusive study of nitrogen starvation-dependent diploid pseudohyphal growth.

## Results

### Identification of proteins that associate with the Kog1/TORC1 complex

**Figure 1:**
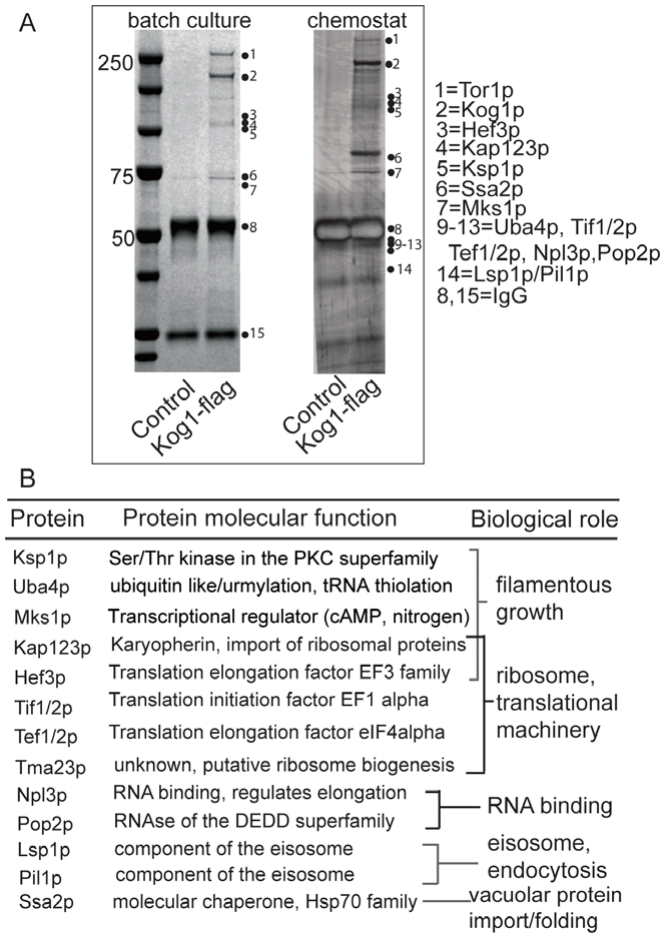
Purified Kog1p/TORC1 associates with proteins involved in the translational machinery, endocytosis, RNA binding and filamentous growth. The Kog1p/TORC1 complex was purified from yeast (CEN.PK genetic background) grown in different nutrient conditions and numerous TORC1-associated proteins were identified. (A) SDS-PAGE analysis of samples from typical purifications of TORC1 from yeast grown in batch cultures containing standard glucose medium (Coomassie blue) or yeast grown in chemostat cultures under glucose-limited conditions (silver stain). Specific bands were cut out and distinct TORC1-associated proteins were identified by mass spectrometry analysis. For clarity purposes, not all proteins/bands identified are named on the gel, but listed in the table (panel B). (B) Organization of putative TORC1-interacting proteins according to known/observed biological function. The identified proteins could be classified broadly under “ribosome and translational machinery”, “endocytosis and eisosomes”, “RNA binding” and “filamentous growth”.

We developed a reliable, efficient method to biochemically isolate TORC1 from yeast grown under different nutrient conditions (see Materials and Methods). Our immunopurification yielded TORC1 containing Kog1p and Tor1p at stoichiometrically similar levels, as well as a number of specifically associated proteins (at non-stoichiometric levels) that do not co-purify in control samples (Figure 1A). A number of these associated proteins were excised from SDS-PAGE gels and identified using standard nano-HPLC/MS/MS methods, resulting in the identification of several new as well as known TORC1 components (Figure 1A). When classified by known function, there appeared to be four broad functional groups of proteins that interact with the yeast TORC1 based on our purification (Figure 1B). These included the translational machinery and ribosome biogenesis (Tef2p, Tif2p, Kap123p and putatively Hef3p, Tma23p), RNA binding proteins potentially related to translation (Npl3p, Caf1p), endocytosis/eisosome (Lsp1p and Pil1p), as well as several proteins involved in filamentous growth.

TORC1 is known to be a major regulator of the translational machinery, RNA regulation and ribosome biogenesis. Many of the proteins we identified in the first three groups had not been studied previously as TORC1 components or TORC1-regulated proteins. We tested the effect of individual deletion mutants of these proteins on yeast growth in the presence of sub-lethal concentrations of rapamycin, in order to determine whether these proteins were potentially genetically linked to the TORC1 pathway. A majority of these proteins showed altered sensitivity to growth in the presence of rapamycin (Table 1). While this does not definitively show that these proteins associate with the TORC1, it suggests that these proteins are potentially linked to the TORC1 pathway. These data provided confidence in our purifications of the TORC1, and the identification of TORC1-interacting proteins. While these proteins are not a focus of this study, they drew our attention to the fourth group of identified proteins.

**Table 1:** Effect of 40 ng/ml rapamycin on the growth of wild type or mutant *S. cerevisiae* (CEN.PK background).

Strain	Resistance to rapamycin	Known biological role of gene
WT	-	
*pil1Δ*	decreased resistance	eisosome
*lsp1Δ*	slightly decreased resistance	eisosome
*tef2Δ*	greatly decreased resistance	translational machinery
*tif2Δ*	no overt phenotype	translational machinery
*npl3Δ*	decreased resistance	RNA binding, elongation
*caf1Δ*	decreased resistance	RNA binding
*tma23Δ*	slightly decreased resistance	ribosome biogenesis
*ssa2Δ*	no overt phenotype	vacuolar member: HSP70 family

This last subset of TORC1-associated proteins identified were all linked to various types of fungal filamentous growth (as described within the yeast genome database). This included Mks1p, Kap123p, Hef3p, Ksp1p and Uba4p. We were surprised to observe so many proteins involved in filamentous growth apparently associate directly with the TORC1. Although prior studies had shown that the TORC1 and rapamycin may be involved in yeast filamentous growth [31], the role of TORC1 in these types of differentiation has not been significantly investigated. Hence, in this study, we decided to focus on the roles of these proteins in relation to their function in filamentous growth and TORC1. Uba4p is a conserved protein that is essential for tRNA thiolation and required for the covalent attachment of a ubiquitin-like protein, Urm1p, to other proteins [32], [33], [34]. Mks1p is a transcriptional regulator involved in nitrogen regulation and lysine biosynthesis [35], [36], [37]. Ksp1p is a member of a stress-activated protein kinase superfamily implicated in fungal filamentous growth [38]. Kap123p is a member of the Karyopherin beta family and is involved in the nuclear import of ribosomal proteins prior to assembly into ribosomes [39]. Hef3p is a paralog of the translation elongation factor EF3. Some of these proteins are fairly well-studied. However, only Ksp1p had already been shown to be directly associated with TORC1 in a recent global analysis study of kinase-kinase interactions [40]. This report did not focus on the role of Ksp1p in fungal invasive/filamentous growth in relation to TORC1. None of the other four proteins reported in this study have been shown before to directly interact with the TORC1, though *uba4* and *mks1* mutants are known to be rapamycin-sensitive and potentially TORC1-regulated [35], [41].

### Identified proteins implicated in filamentous growth directly associate with TORC and exhibit rapamycin-associated phenotypes when deleted

**Figure 2:**
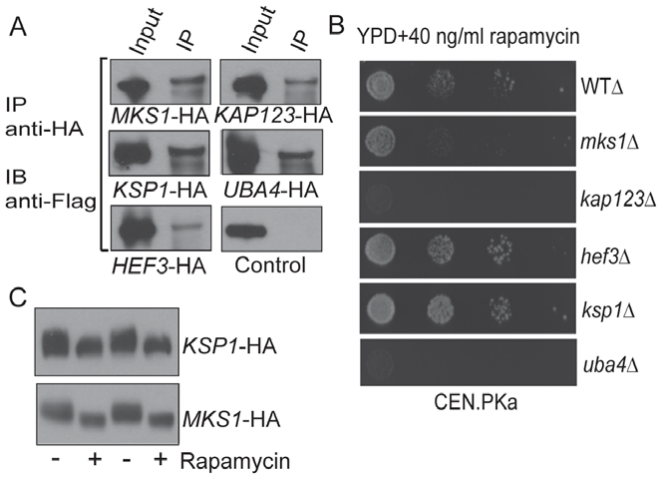
Identified proteins involved in filamentous growth interact directly with the TORC1. (A) Identified proteins listed to be involved in filamentous growth were HA-epitope tagged at the C-terminus in strains expressing *KOG1*-FLAG, and immunoprecipitated using HA-epitope specific antibodies. *KOG1*-FLAG was detected by Western blotting, using an anti-flag antibody. Control IPs were performed using *KOG1*-FLAG strains without HA-tagged proteins. (B) The effect of sub-lethal concentrations of rapamycin (40 ng/ml) on the growth of wild type (WT) or mutant yeast (CEN.PK background) was measured after 72 hours of growth. Mutants of the identified proteins showed increased or decreased growth in the presence of rapamycin. (C) Yeast cells (CEN.PK background) expressing *KSP1*-HA or *MKS1*-HA were grown in YPD in the presence or absence of 100 nM rapamycin for 25 min, proteins were extracted under denaturing conditions and their SDS-PAGE migration was assayed by Western blotting (anti-HA) in order to observe rapamycin-dependent mobility changes.

In order to confirm that the identified subset of proteins putatively involved in yeast filamentous growth could indeed associate with the TORC1 and were in the TORC1 pathway, we used a combination of biochemical, genetic and pharmacological methods. Since the interacting proteins were originally identified when *KOG1*-FLAG was immunopurified using a FLAG antibody, Mks1p, Kap123p, Hef3p, Ksp1p and Uba4p were tagged with a C-terminal HA epitope tag in yeast strains expressing *KOG1*-FLAG. Subsequently, these proteins were immunoprecipitated using an HA antibody, and the co-precipitation of Kog1p was tested by detecting the FLAG epitope on Kog1p (Figure 2A). Kog1p was consistently found to be associated with a small fraction of each of these five indicated proteins (Figure 2A), strongly suggesting that they all are at least transiently and non-stoichiometrically associated with TORC1. Thus, immunopurification of Kog1p identified these proteins, and individual immunopurifications of these proteins co-precipitated Kog1p.

Next, individual deletion mutants of these five genes were then constructed, and the growth of these yeast strains were tested on plates containing standard glucose rich media (Figure 2B) as well as minimal glucose medium (not shown) in the presence of sub-lethal concentrations of rapamycin. Notably, these mutants exhibited significant growth phenotypes (compared to wild type) in response to sub-lethal rapamycin concentrations, suggesting that they were all either potentially regulated by TORC1, or function upstream of TORC1 (Figure 2B). Interestingly, the different mutants displayed a wide variety of responses to rapamycin. Some mutants such as *uba4Δ* (described earlier in [32]) and *kap123Δ* showed dramatically reduced growth in the presence of rapamycin. In contrast, *ksp1Δ* and *hef3Δ* (to a far lesser extent) showed slightly increased growth in the presence of rapamycin. Interestingly, *mks1Δ* mutants showed decreased resistance to rapamycin, in contrast with an earlier report [26]. However, on several occasions, particularly with strains that had been maintained on drug-free plates for several weeks, we have observed that *mks1Δ* strains show a tendency to revert to an increased rapamycin-resistance phenotype (Figure S2, compared to Figure 2B). Both results only further confirm the chemical genetic link between Mks1p and the TORC1. Finally, two of these proteins, Mks1p and Ksp1p, displayed a rapamycin-dependent mobility shift during SDS-PAGE (Figure 2C), as has also been shown earlier [26], [36], suggesting their phosphorylation in a TORC1-dependent manner. Collectively, these data demonstrate that Mks1p, Kap123p, Hef3p, Ksp1p and Uba4p each can directly associate with TORC1, and that their biological functions are likely regulated in a TORC1-dependent manner.

We also tested the rapamycin sensitivity of mutants of these proteins constructed in Σ1278b strains of yeast, which are widely used in a variety of yeast invasive growth studies. The overall trend was similar, with *mks1Δ*, and *uba4Δ* showing greatly decreased resistance to rapamycin, and *ksp1Δ* and *hef3Δ* strains showing increased resistance to rapamycin (Figure S1). However, there are some strain-specific differences between CEN.PK and Σ1278b genetic backgrounds, notably for *kap123Δ* mutant strains. *kap123Δ* mutants from a Σ1278b background showed increased sensitivity to rapamycin, but were not as sensitive as *kap123Δ* strains from a CEN.PK background (Figure S1 compared to Figure 2B) .

Thus, we have identified two novel TORC1-associated proteins (Kap123p and Hef3p), with *kap123Δ* mutants being particularly rapamycin-sensitive. We also show that Mks1p and Uba4p (both known to exhibit rapamycin phenotypes [35], [41]) can directly associate with the TORC1, suggesting they are TORC1 targets or partners. We also independently confirm Ksp1p to be both associated with TORC1 and phosphorylated in a TORC1/rapamycin-dependent manner. Collectively, these data suggest that these proteins are likely regulated by the TORC1 either directly or through directly-associated proteins, or function upstream of TORC1.

### Nitrogen starvation-dependent diploid pseudohyphal growth and other forms of invasive growth may be differentially regulated

**Figure 3:**
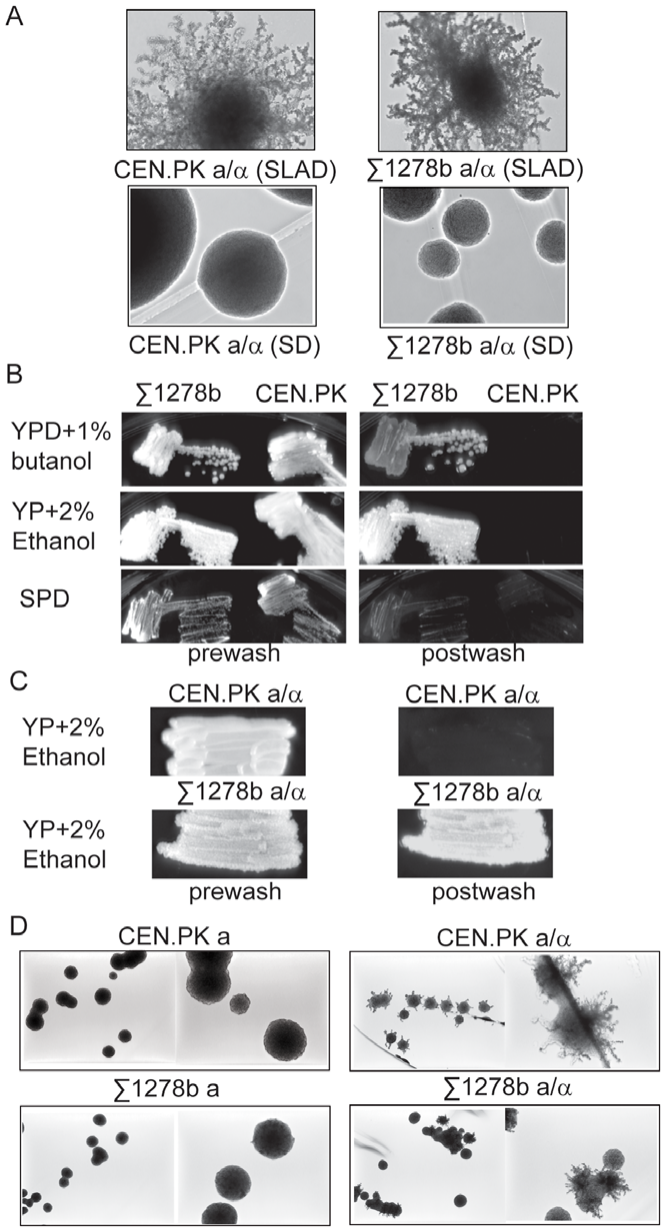
Nitrogen starvation-dependent diploid pseudohyphal growth and other haploid invasive growth may be differentially regulated. Diploid CEN.PK and Σ1278b background strains show robust nitrogen-starvation dependent pseudohyphal growth but haploid CEN.PK strains do not show other invasive growth. (A) Diploid yeast cells from a CEN.PK or a Σ1278b background were grown on nitrogen-starved SLAD plates or on standard complete nitrogen plates (SD) containing 1% glucose as a carbon source. Pseudohyphal growth was observed and colonies were imaged after growth for 2 weeks. (B) Haploid yeast cells from a CEN.PK or a Σ1278b background were grown under a variety of conditions known to induce invasive growth, including YP Dextrose+butanol, YP+ethanol, and proline as a nitrogen source (SPD). Invasive growth was tested after 72 hours by washing the plates under a steady stream of water, with plates further washed for 3 minutes after all CEN.PK background yeast were washed off. (C) Diploid yeast cells from a CEN.PK or a Σ1278b background were grown on YP+Ethanol and invasive growth was observed after 72 hours and subsequent washing of plates. (D) Nitrogen starvation-dependent pseudohyphal growth was assayed in haploid yeast cells from CEN.PK or Σ1278b backgrounds grown on SLAD agar, and compared to diploid pseudohyphal growth. Cells were grown on SLAD agar plates for 2 weeks and then imaged. The left panels of each strain show 4× magnification images, and the right panel show 10× images.

The vast majority of the studies on the various types of filamentous and invasive growth in yeast have been conducted using the Σ1278b strain background, where diploid pseudohyphal growth as well as other types of haploid invasive growth were first demonstrated [3], [5], [6]. However, yeast strains from the Σ1278b background readily undergo differentiation into these filamentous forms under a variety of conditions. Therefore it remains unclear if these different types of differentiation caused by glucose limitation, nitrogen limitation or other environmental factors are essentially the same, or are sensed by different pathways that finally converge on the PKA, MAPK and Snf1 nodes. Additionally, our TORC1 purifications that isolated several proteins involved in filamentous growth were carried out in yeast from a CEN.PK genetic background which has not been tested for filamentous growth. To this end, we compared the various types of nutrient-dependent differentiation of diploid and haploid yeast from a prototrophic Σ1278b background with a prototrophic CEN.PK background. Under nitrogen starvation conditions, diploid Σ1278b yeast readily formed pseudohyphae (Figure 3A). Interestingly, diploid CEN.PK yeast also showed extremely robust and extensive pseudohyphae formation when grown under nitrogen-restricted conditions, similar to those formed by yeast from the Σ1278b background (Figure 3A). However, when grown on various other conditions which induce strong invasive/filamentous growth in haploid Σ1278b strains (including growth on non-fermentable carbon sources, the presence of alcohols, and elevated cAMP levels), haploid CEN.PK yeast did not show any invasive growth (Figure 3B). This was in stark contrast to the robust invasive growth seen in Σ1278b background strains under various conditions (Figure 3B). These observations on haploid invasive growth were carried out using standard invasive growth assays wherein plates streaked with the respective strains of yeast were washed under a steady stream of water. All plates were washed until all non-invasive CEN.PK yeast were removed, and further washed for three more minutes. This removes all cells that have not even weakly invaded the agar, but may not remove cells that weakly invade agar. This was done to particularly contrast invasive growth in the Σ1278b background strains with the CEN.PK background strains.

We further determined if these observations in the CEN.PK strain background were due to some effect of yeast ploidy and/or nutrient conditions. While diploid yeast from a Σ1278b background continued to invade the agar under various conditions (such as plates containing ethanol as a carbon source (Figure 3C) or high levels of cAMP (not shown)), diploid CEN.PK background yeast did not show any invasion of the agar (Figure 3C). Finally, nitrogen starvation-dependent pseudohyphal growth has primarily been observed only in diploid yeast (described in [3], [11]). For completeness, we tested the growth of haploid yeast from a CEN.PK or Σ1278b genetic background under nitrogen-depleted conditions. Expectedly, haploid yeast from both backgrounds did not show any significant pseudohyphal differentiation under nitrogen restriction (Figure 3D), while diploid yeast from both backgrounds readily did. These data likely rule out any differences due to ploidy or growth conditions tested, and suggest that both genetic backgrounds respond strongly to nitrogen starvation, but only the Σ1278b background strains respond to diverse stimuli related to carbon sources. Collectively, these data suggest that CEN.PK strains undergo differentiation only under nitrogen-depleted conditions and not other nutritional or environmental stresses. Moreover, these observations may suggest intrinsic differences in the pathways that initially sense and respond to nitrogen starvation in diploid yeast resulting in pseudohyphal growth, compared to other nutritional stresses resulting in haploid or diploid invasive growth.

### TORC1 may not play a major role in nitrogen-independent haploid invasive growth

**Figure 4:**
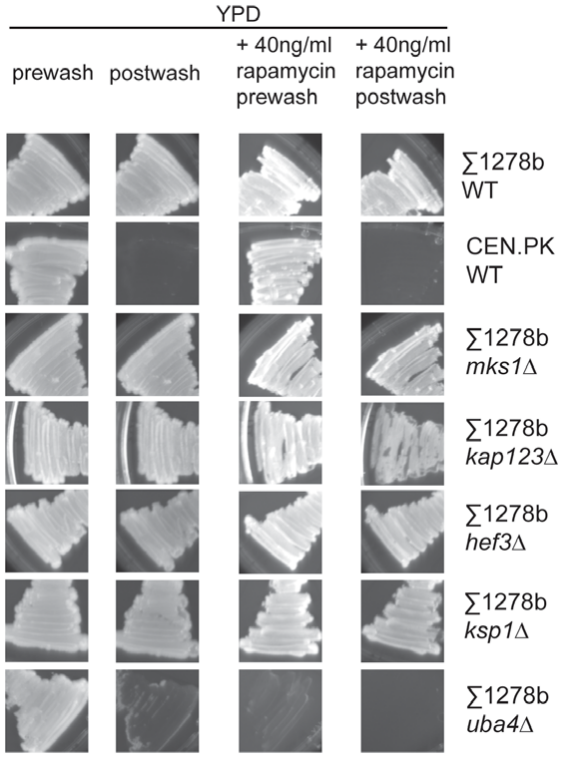
The TORC1 or TORC1-associated proteins may not regulate haploid invasive growth. Wild type or mutant strains of *S. cerevisiae* from a Σ1278b background were grown on rich YP+2%Dextrose plates which stimulated strong invasive growth on solid agar plates, including in the presence of sub-lethal rapamycin concentrations (40 ng/ml). Non-invasive CEN.PK background strains were used as a control. Invasive growth washing assays were performed after 72 hours (for YPD plates) or 5 days (YPD+rapamycin plates). Plates were washed continuously for 3 minutes after all non-invasive yeast streaks (CEN.PK genetic background) were washed away. Several other growth conditions promoting invasive growth were also tested for wild type and mutant strains are shown in Figure S3. *uba4Δ* strains showed negligible growth in the presence of rapamycin, hence invasive growth could not be tested under those conditions. *kap123*Δ strains (and *mks1Δ* strains to a lesser extent) exhibited reduced growth (compared to WT strains) in the presence of rapamycin. However, substantial growth can be observed at the end of 5 days, and both strains continue to show haploid invasive growth. Invasive growth under more stringent wash conditions with extensive rubbing of streaked cells during washing (to remove more weakly invasive cells) are shown in Figure S4.

Our purifications of the TORC1 identified proteins involved in different types of yeast filamentous growth that were associated as part of the complex. However, both the CEN.PK and Σ1278b yeast strains show robust nitrogen-dependent diploid pseudohyphal growth, but the CEN.PK strain does not show haploid invasive growth under any of the other conditions. Therefore, we next attempted to ascertain if the TORC1 pathway and/or any of the identified TORC1-associated proteins played a role in nitrogen-independent invasive growth. First, we tested invasive growth in haploid, prototrophic Σ1278b background yeast strains under a variety of conditions, in the presence or absence of sub-lethal concentrations of the TORC1 inhibitor rapamycin. Yeast continue to show robust invasive growth even under partial TORC1 inhibition by rapamycin (Figure 4 and Figures S3 and S4), except when grown with ethanol as the sole carbon source (yeast from the Σ1278b genetic background appear to be hypersensitive to rapamycin and grow extremely poorly on ethanol in the presence of rapamycin, Figure S3, and hence invasive growth under these conditions cannot be tested). These data suggest that the TORC1 pathway may not significantly regulate haploid invasive growth. Consistent with this observation, most deletion mutants of proteins we identified as TORC1-associated continued to show significant invasive growth under these conditions (Figure 4 and Figures S3 and S4). *ksp1Δ* deletion mutants showed slightly diminished invasive growth only under some invasive growth conditions (Figure 4 and Figure S3 and S4). *kap123Δ* strains also show a small decrease in invasive growth (Figure 4 and Figures S3 and S4). Only *uba4Δ* mutants were completely unable to invade their growth media when grown on rich YPD plates (Figure 4), or under elevated cAMP levels or in the presence of alcohols such as butanol (Figures S3 and S4), suggesting an important role for Uba4p in various types of yeast filamentous growth, some of which may be TOR-independent. None of these mutants, except the *uba4Δ* mutant, showed any significant defects in invasive growth when grown on a non-fermentable carbon source (ethanol) requiring respiratory function (Figures S3 and S4). Figure S3 shows the extent of invasive growth observed after washing plates under a steady stream of water, which only washes off non-invasive cells or very weakly invasive cells. This allowed us to observe smaller defects in invasive growth in *ksp1Δ* and *kap123Δ* strains. Figure S4 shows invasive growth in these strains after very stringent washing with extensive rubbing (using a gloved finger) of the streaked strains. This also removes all more weakly invasive yeast cells, leaving only the cells that have deeply invaded the agar. Under these conditions, the absence of deep invasive growth of *uba4Δ* strains even when grown on plates with ethanol as the sole carbon source is readily apparent. *uba4Δ* mutants in particular, (as well as *kap123Δ* and *mks1Δ* mutants to a far lesser extent), grew poorly in the presence of rapamycin, hence our interpretation for these mutants are primarily based on results from various invasive growth media in the absence of rapamycin. The slower growth of *kap123Δ* and *mks1Δ* in the presence of rapamycin is more apparent in Figure S4. In other studies, Ksp1p and Mks1p have been shown to play a role in haploid invasive growth [36], [38]. Some of this might be in part because of differences between the prototrophic Σ1278b strains used here and the auxotrophic strains used in those studies, which are known to show less robust filamentous growth and may be particularly sensitive to any inputs that regulate any kind of filamentous growth (as described in the history of Σ1278b and notes on other Σ1278b derivative sets provided by the Fink lab, available on the yeast genome wiki: http://wiki.yeastgenome.org/index.php/History_of_Sigma). The smaller effects on haploid invasive growth observed in *ksp1Δ* strains observed in this study are partially consistent with earlier work [38]. In summary, TORC1 inhibition (with rapamycin) did not appear to significantly inhibit haploid invasive growth in all conditions tested. Of all the TORC1-associated proteins identified, only *uba4Δ* strains showed severe defects in haploid invasive growth under many (but not all) invasive growth conditions tested.

### TORC1 may regulate nitrogen-dependent pseudohyphal growth in part through the various TORC1 associated proteins identified

**Figure 5:**
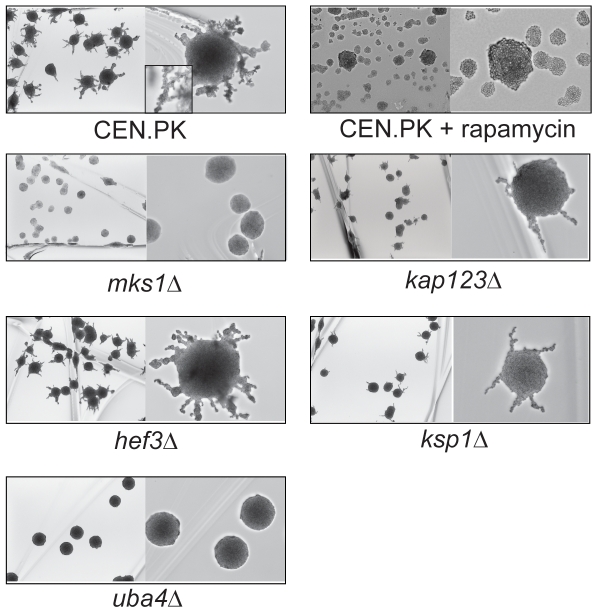
TORC1-associated proteins are involved in nitrogen-dependent pseudohyphal growth in diploid yeast. Wild type or mutant CEN.PK background strains of yeast were grown in nitrogen-starved SLAD medium in the presence or absence of sub-lethal concentrations of rapamycin, and pseudohyphal growth was measured after 2 weeks of growth. Two panels are shown for each strain; the left panel shows a 4× magnification image of a broad field of colonies, and the right panel shows a 10× magnification (with a further 1.5× digital magnification) of one or two colonies with more extensive pseudohyphal development seen in that specific strain. The inset in the CEN.PK panel shows a higher magnification (10×+4× digital magnification) depicting the elongated, extending cells seen typically with pseudohyphal growth.

We next tested diploid pseudohyphal growth under nitrogen-depleted conditions in strains containing deletions of the identified TORC1 components. Wild type yeast from a CEN.PK background showed strong pseudohyphal growth under nitrogen starvation (Figure 5). This phenotype was completely absent when the cells were grown in the presence of sub-lethal concentrations of rapamycin (tested at 15 and 40 ng/ml, and shown for 40 ng/ml). Previous work using a Σ1278b background strain had shown that rapamycin inhibits filamentous growth, and protein phosphatase Sit4p, acting downstream of TORC1, played a role in controlling pseudohyphal growth [31]. Importantly, in our experiments, diploid deletion mutants of *mks1Δ*, *kap123Δ*, *hef3Δ*, *ksp1Δ*, and *uba4Δ* strains in the CEN.PK background all showed altered levels of diploid pseudohyphal growth upon nitrogen-depletion. In multiple experiments, nearly all the wild type cells streaked out on nitrogen-depleted SLAD medium plates showed robust, extensive pseudohyphal growth (Figure 5). With *ksp1Δ* and *kap123Δ* strains streaked out to very similar densities, pseudohyphal growth was observed in a smaller number of colonies, with very few colonies showing elaborate, well-developed pseudohyphae (Figure 5). In *mks1Δ* and *uba4Δ* strains from a CEN.PK background, diploid pseudohyphal growth was significantly reduced (*mks1Δ*) or completely absent (*uba4Δ*) when grown under the same conditions (Figure 5). The phenotype for *hef3Δ* strains was a little less obvious. A few cells showed elaborate diploid pseudohyphal growth, but while most cells showed significant pseudohyphae formation, there appeared to be a small qualitative difference in the number and extent of pseudohyphae formation (Figure 5). For this strain, it was not possible to obtain a qualitative conclusion from multiple experiments.

We also repeated these same experiments in diploid wild type and mutant strains from a Σ1278b background. These strains largely showed a similar trend of phenotypes as observed in the CEN.PK background (Figure S5 and summarized in Table 2), with some strain-specific differences. Interestingly, *kap123Δ* strains (which exhibit reduced relative rapamycin sensitivity in the Σ1278b background compared to the CEN.PK background, Figure S1), correspondingly showed a less severe pseudohyphal growth defect in this genetic background, consistent with our observations thus far (Figure S5). Surprisingly, the *mks1Δ* strain in this genetic background also showed more pseudohyphal growth compared to the CEN.PK background (where pseudohyphal growth was almost completely absent), but showed considerably less pseudohyphal growth than wild type Σ1278b background strains. Like the CEN.PK background, *hef3Δ* mutant strains in the Σ1278b background had the least pronounced phenotype, with small qualitative, but hard to visualize, differences in pseudohyphal growth. Collectively, our data indicate that the TORC1, in part through these novel TORC-associated proteins, might function to regulate specifically nitrogen starvation-induced pseudohyphal growth.

**Table 2:** Summary of strain specific effects on nitrogen starvation-dependent pseudohyphal growth.

Gene	Background	Pseudohyphal growth
*mks1*	CEN.PK	Greatly decreased or absent
*kap123*	CEN.PK	Greatly decreased
*hef3*	CEN.PK	Unclear/small decrease
*ksp1*	CEN.PK	Decreased
*uba4*	CEN.PK	Absent
*mks1*	Σ1278b	Decreased
*kap123*	Σ1278b	Decreased
*hef3*	Σ1278b	Unclear/small decrease
*ksp1*	Σ1278b	Decreased
*uba4*	Σ1278b	Absent

### *FLO11* expression is reduced in mutant strains primarily under nitrogen-restricted conditions

**Figure 6:**
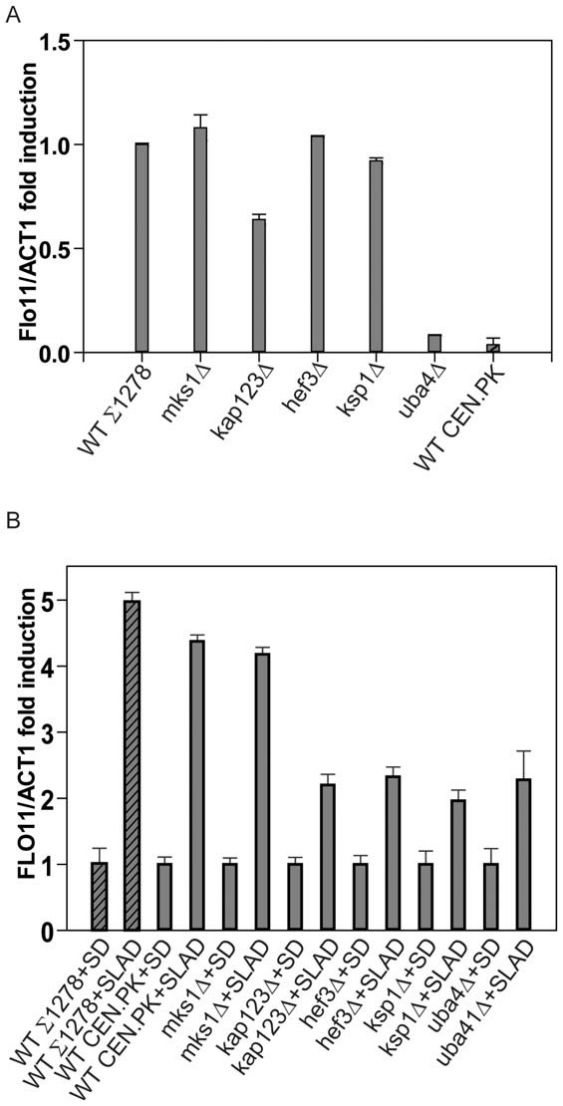
Induction of *FLO11* gene expression is decreased in diploid mutant strains under nitrogen starvation. A) The relative *FLO11* gene expression of haploid mutant strains in a Σ1278b background and wild type Σ1278b strains were compared (with wild type strains having a relative expression of one) from cells grown in YPD using quantitative RT-PCR. *FLO11* expression in haploid yeast from a CEN.PK background was also compared to the Σ1278b strain. B) Quantitative RT-PCR measurements of relative *FLO11* gene expression in wild type diploid yeast (CEN.PK genetic background) grown in normal media (SD) or nitrogen-starved media (SLAD) were carried out.

The various pathways that regulate invasive or pseudohyphal growth in yeast appear to converge on the promoter of the *FLO11* gene (under both nitrogen starvation and varied carbon sources [11], [42], [43]). The cAMP, MAPK and Snf1 pathways all induce the expression of *FLO11*, which encodes a cell surface protein critical for the differentiation of unicellular yeast into these invasive/pseudohyphal forms [11], [42], [43]. In order to obtain a more precise, quantitative measure of the extent of regulation of *FLO11* gene expression in wild type strains or mutant strains discussed in this study (particularly under conditions that promote diploid pseudohyphal growth), we carried out quantitative real-time PCR measurements of *FLO11* gene induction from cells grown in a variety of conditions. First, *FLO11* gene expression was measured in haploid wild type strains from a CEN.PK background, and in wild type and mutant strains from a Σ1278b background when grown in standard YPD medium (where Σ1278b strains show substantial invasive growth). Relative levels of *FLO11* gene expression were compared to wild type Σ1278b background strains (Figure 6A). Wild type strains from a CEN.PK background had relatively low relative levels of *FLO11*, consistent with the absence of haploid invasive growth observed in these strains (Figure 6A). Also consistent with their invasive growth phenotypes, *uba4Δ* strains showed very low relative levels of *FLO11* gene expression. *kap123Δ* strains showed a slightly lower relative *FLO11* gene levels, again consistent with observed invasive growth phenotypes. The other mutant strains showed substantial *FLO11* expression (Figure 6A). To test the induction of *FLO11* under conditions of nitrogen deprivation, diploid yeast strains were grown in standard media containing normal nitrogen levels (SD) or transferred to nitrogen-depleted media (SLAD). Cells were collected under both conditions and relative *FLO11* gene expression was measured. When transferred to nitrogen-depleted medium, wild type yeast from both genetic backgrounds showed a 4–5 fold increase in *FLO11* mRNA levels (Figure 6B), suggesting a strong response to nitrogen starvation that can lead to pseudohyphal growth. Importantly, mutant strains *kap123Δ*, *hef3Δ*, *ksp1Δ* and *uba4Δ* all showed significantly lower levels of relative *FLO11* gene induction (Figure 6B). Surprisingly, the *mks1Δ* strain continued to show substantial *FLO11* gene induction, although these mutants showed very little pseudohyphal growth in the CEN.PK background (Figure 5). Collectively, these *FLO11* expression data are consistent with our observations that the TORC1 and the identified TORC1-associated proteins play critical roles in nitrogen-dependent diploid pseudohyphal growth.

## Discussion

The data presented in this study provide insight into the broader roles of the TORC1 in response to low nitrogen levels. Our data show that the TORC1-interacting proteins identified in this study are involved in diploid pseudohyphal growth (Mks1p, Kap123p, Ksp1p, Hef3p and Uba4p). Interestingly, these proteins show different responses to rapamycin treatment. While the deletion of some of these proteins (Ksp1p, Hef3p) increases growth in the presence of rapamycin, the deletion of others (Mks1, Kap123p, Uba4p) decreases growth in the presence of rapamycin. This is consistent with a more nuanced response of TORC1-regulated proteins under conditions of TOR kinase inhibition, as well as potential TOR kinase activity-independent roles for TORC1 under conditions such as nitrogen starvation. Interestingly, and consistent with our own experimental observations, we examined a large data set from a genome-wide study of mutants with altered filamentous growth [44], and the deletion or over-expression of several of these identified genes also increased or decreased resistance to rapamycin. Collectively, our data suggest a key role for the TORC1 pathway in responding to nitrogen starvation and inducing filamentous growth in diploid yeast. This also supports the idea that nitrogen starvation-induced inhibition of TOR kinase activity potentially results in the activation of a set of proteins along with the inactivation of others. Such modulation could confer TORC1 with the ability to not only respond to increased nitrogen levels, but also regulate responses to low nitrogen levels as well.

The effects of mutants of the core components of TORC1 themselves (such as Kog1p, Tor1p, Tor2p, Lst8p) on yeast filamentous growth themselves are unclear and much harder to study, since mutants of these proteins are severely compromised for normal growth. Moreover, it remains an open question how important TORC1 regulation (by phosphorylation) is for the biological activity of the proteins identified in this study. However, it is particularly challenging to identify TORC1-specific or TORC1 effector-specific phosphorylation sites, particularly in proteins like Mks1p, Ksp1p, Kap123p and Hef3p which contain a large number of Ser/Thr residues predicted to be kinase target sites. We hope future studies will reveal the role and importance of TORC1-dependent phosphorylation of these proteins during yeast filamentous growth and other biological functions.

Under conditions that promote haploid invasive growth, the trends we observe are suggestive but not conclusive for a relatively lesser role for TORC1 in these processes. Rapamycin did not inhibit haploid invasive growth in the Σ1278b background under most conditions. Additionally, only *uba4Δ* strains showed severe haploid invasive growth defects under many (but not all) conditions tested. Two other strains, *kap123Δ* and *ksp1Δ* show relatively small defects in haploid invasive growth. Finally, *FLO11* gene expression (encoding the cell-surface flocculin essential for yeast invasion or pseudohyphae formation) is significantly induced in most mutant strains (except *uba4Δ* and to a lesser extent *kap123Δ* strains, entirely consistent with experimental observations) from a Σ1278b genetic background. Part of the ambiguity in conclusively ruling out a role of the TORC1 in haploid invasive growth is due to its strong overlap with the PKA pathway. Several studies now describe the extent of TORC1 and cAMP/PKA pathway cross-communication and coordination of cellular responses to nutrients [45], [46], [47], [48], [49], with a recent study outlining how TOR controls PKA activity towards specific substrates [45]. This study also shows altered phosphorylation states of cAMP/PKA phosphorylation sites on two proteins discussed in our study (Mks1p and Ksp1p) upon rapamycin treatment/TORC1 inhibition [45], suggesting intimate cross-talk between the two pathways. Thus, while the final outcome of all forms of diploid and haploid yeast filamentous may be PKA regulated, the TORC1 may be more specific to diploid pseudohyphal growth. Consistent with this idea is the observation that constitutive cAMP signaling can bypass potential TORC1-mediated regulation of pseudohyphal growth [31]. We speculate that yeast differentiate diploid pseudohyphal growth from other filamentous behavior, not in terms of the phenotypic output, but in terms of the signaling pathway that receives and responds to the input. Future work can specifically outline the interplay between TORC1 and the proteins identified in this study, during pseudohyphal growth and other functions.

The PKA and MAPK pathways are the key mediators of various forms of filamentous growth and cellular differentiation in yeast, with the nutrient sensing Snf1 complex being one key regulatory node for regulating this developmental switch. Yet, yeast appear to be exquisitely tuned to respond to a diverse array of environmental inputs (from low nitrogen to absence of fermentable carbon sources) that induce filamentous behavior. This leaves open the possibility that these different inputs could be sensed by different pathways that finally signal through the cAMP/PKA and Snf1 nodes. Our study suggests that the TORC1 may sense and regulate some responses to low nitrogen levels, in part by bringing together a diverse biochemical complex of proteins with different functions, which control yeast differentiation. Consistent with our data, previous observations demonstrated that mutants of Snf1, which is critical for nitrogen starvation-dependent pseudohyphal growth, are highly rapamycin-sensitive and TORC1-dependent [16], [17], [50].

It remains a serendipitous observation that yeast from a CEN.PK genetic background do not show any haploid invasive growth under a variety of conditions, but show very robust nitrogen starvation-dependent pseudohyphal differentiation. It is possible that this is due to the fact that many yeast strains from a CEN.PK genetic background have a single point mutation close to the C-terminus of the adenylate cyclase, Cyr1, which delays glucose-induced loss of stress resistance [51], but does not seem to cause any major defects in the growth of this strain or other cAMP-dependent responses. No other obvious polymorphisms in the TOR pathway or the cAMP pathways were found in this strain based on existing polymorphism data [52], [53]. However, the CEN.PK background strain of yeast seems exquisitely sensitive to nitrogen levels, and shows strong nitrogen-dependent pseudohyphal development. Thus, it may represent a useful strain to specifically investigate the dedicated sensory systems that respond to nitrogen starvation and induce pseudohyphal growth in yeast.

## Materials and Methods

### Yeast strains, deletions and epitope tagging

The prototrophic CEN.PK strain background [54] was used for biochemical purification of the TORC1, and for all biochemical purification and interaction experiments. Invasive growth assays were carried out in a prototrophic CEN.PK genetic background strain (MATa or MATa/MATα) and in a prototrophic Σ1278b genetic background strain (MATa or MATa/MATα). Pseudohyphal growth under nitrogen starvation was tested in prototrophic CEN.PK background strains (MATa/MATα) and Σ1278b background strains (MATa/MATα). The respective mutant diploid strains (CEN.PK or Σ1278b background) were created by deleting the specified gene in MATa and MATα strains and subsequently mating them together to obtain the diploid homozygous deletion strain. All strains used in this study are listed in Table 3. All gene deletions were carried out by standard PCR based gene deletion [55] and oligonucleotides used in generating deletions are listed in Table 4. Deletions were confirmed using PCR. C-terminal epitope tagging of specific genes was also carried out using a PCR based gene tagging method, and oligonucleotides used to make these strains are listed in Table 4. Tagging was confirmed by PCR as well as western blotting for the epitope tag used.

**Table 3:** *S. cerevisiae* strains used in this study.

Background	Strain genotype	Source
CEN.PK	MAT a, MAT α, MAT a/α	P. Kotter
Σ1278b (F1322)	MAT α	Fink lab
Σ1278b (MB100)	MAT a	Fink lab via Brandriss lab
Σ1278b	MAT a/α	This study
CEN.PK	MAT α *KOG1*-Flag::KanMX4	This study
CEN.PK	MAT α *KOG1*-Flag::KanMX4 *MKS1*-HA::NAT	This study
CEN.PK	MAT α *KOG1*-Flag::KanMX4 *KAP123*-HA::NAT	This study
CEN.PK	MAT α *KOG1*-Flag::KanMX4 *HEF3*-HA::NAT	This study
CEN.PK	MAT α *KOG1*-Flag::KanMX4 *KSP1*-HA:NAT	This study
CEN.PK	MAT α *KOG1*-Flag::KanMX4 *UBA4*-HA::NAT	This study
CEN.PK	MAT a *mks1*::NAT MAT α *mks1*::NAT	This study
CEN.PK	MAT a *kap123*::NAT MAT α *Kap123*::NAT	This study
CEN.PK	MAT a *hef3*::NAT MAT α *hef3*::NAT	This study
CEN.PK	MAT a *ksp1*::NAT MAT α *ksp1*::NAT	This study
CEN.PK	MAT a *uba4*::HYG MAT α *uba4*::NAT	This study
Σ1278b	MAT a *mks1*::NAT MAT α *mks1*::NAT	This study
Σ1278b	MAT a *kap123*: MAT α *kap123*::NAT	This study
Σ1278b	MAT a *hef3*: MAT α *hef3*::NAT	This study
Σ1278b	MAT a *ksp1*::NAT MAT α *ksp1*::NAT	This study
Σ1278b	MAT a *uba4*::HYG MAT α *uba4*::NAT	This study
CEN.PK	MAT α *mks1*::NAT	This study
CEN.PK	MAT α *kap123*::NAT	This study
CEN.PK	MAT α *hef3*::NAT	This study
CEN.PK	MAT α *ksp1*::NAT	This study
CEN.PK	MAT α *uba4*::HYG	This study
Σ1278b	MAT α *mks1*::KanMX4	This study
Σ1278b	MAT α *kap123*::KanMX4	This study
Σ1278b	MAT α *hef3*::KanMX4	This study
Σ1278b	MAT α *ksp1*::KanMX4	This study
Σ1278b	MAT α *uba4*::KanMX4	This study
CEN.PK	MAT a *pil1*::NAT	This study
CEN.PK	MAT a *lsp1*::NAT	This study
CEN.PK	MAT a *tif2*::NAT	This study
CEN.PK	MAT a *tef2*::NAT	This study
CEN.PK	MAT a *npl3*::NAT	This study
CEN.PK	MAT a *tma23*::NAT	This study
CEN.PK	MAT a *ssa2*::NAT	This study

**Table 4:** Oligonucleotides used in this study for gene tagging or deletion.

Oligonucleotide	Sequence
*KOG1-tag 5′*	*AAATATCTACAAGTGTGAAGACGAGAGAATTGATTATTTT CGGATCCCCGGGTTAATTAA*
*KOG1-tag 3′*	*TTTGCAGCTAAATGAAAGAAAAAAAAAGAAATGGCACATA GAATTCGAGCTCGTTTAAAC*
*mks1Δ-5′*	*TTCCTAATTA TTCTCTAATC CTAATAAAAA AAAAGAACTG CGGATCCCCGGGTTAATTAA*
*mks1Δ-3′*	*AGAACTTTAAATACTGTATCTGATTTATTTAACTTAGTAA GAATTCGAGCTCGTTTAAAC*
*kap123Δ-5′*	*CTGAGGGACG AAAAACACTT TTTTAGTATC AAGTAGTATA CGGATCCCCGGGTTAATTAA*
*kap123Δ-3′*	*TTATCGAAACAGACGAGAATAAAAAATGGTTTTAAAAAAA GAATTCGAGCTCGTTTAAAC*
*hef3Δ-5′*	*CCTTTTAAAC CAAAAATAAA TAAAAAAAAG GAATCACAAA CGGATCCCCGGGTTAATTAA*
*hef3Δ-3′*	*ATATAAGTTACTCACTGAAAGAGATGTACTCGACTTCAAA GAATTCGAGCTCGTTTAAAC*
*ksp1Δ-5′*	*GTTAGTGCAA TATTTTTTTC TTACAATTTT TTGAAACTCG CGGATCCCCGGGTTAATTAA*
*ksp1Δ-3′*	*AAGAAAATAATAAGCAACATAACAGAGGGAATAGGTGCGC GAATTCGAGCTCGTTTAAAC*
*uba4Δ-5′*	*GCCGTTGACT GCAAAAGGAA GTAAATAGAA GTCAATAACA CGGATCCCCGGGTTAATTAA*
*uba4Δ-3′*	*AAATAAAGTTACATATACACGTTATACATGTATAGGTCAA GAATTCGAGCTCGTTTAAAC*
*MKS1-tag 5′*	*GGAAGCACTGGGGCGTAAGACGAGTAATGGAGGGCGAATA CGG ATC CCC GGG TTA ATT AA*
*KAP123-tag 5′*	*AATTGTTGCTCAAAATCCGGTTTTAGCTGCCGTCATTGCT CGG ATC CCC GGG TTA ATT AA*
*HEF3-tag 5′*	*AGAGATGGGTGATGAATACGTTTCTTCTGATGAAGATTTT CGG ATC CCC GGG TTA ATT AA*
*KSP1-tag 5′*	*ACAATACAAGAATAATTGGTTAATTTTACAGCAACAAGAC CGG ATC CCC GGG TTA ATT AA*
*UBA4-tag 5′*	*CAAATACATAGACGATATTGATCAAACCATTCCTAAATAT CGGATCCCCGGGTTAATTAA*

### Yeast growth and culture

Yeast cells were grown in a variety of conditions in either standard batch cultures in typical rich media (YPD: yeast extract, peptone, 2% dextrose) or nutrient-restricted chemostat cultures containing standard defined glucose media as described earlier [56]. Yeast strains were also grown/streaked out on various solid agar media including low nitrogen media (synthetic low ammonium sulfate and dextrose or SLAD) and synthetic low proline and dextrose or SPD (without any amino acid supplementation), Yeast extract/Peptone/2% Ethanol, Yeast extract/Peptone/Dextrose, Yeast extract/Peptone/Dextrose+1% Butanol, (media compositions as described in other studies [3], [5]) or YPD+cAMP in order to study invasive or pseudohyphal growth. Constitutively high cAMP levels were maintained on plates by adding 0.2 mM concentrations of the PDE hydrolysis resistant, membrane permeable cAMP analog, SP-8-CPT-cAMPS [57]. Haploid invasive growth on various types of solid agar plates was observed using a standard plate washing assay for invasive growth, where a steady, strong stream of water is passed over plates streaked with yeast, washing away cells that have not invaded the agar, as described earlier [3]. In our experiments, we performed this assay in two ways. In brief, cells were streaked on the described plate and allowed to grow for 72 hours, after which they were washed extensively in a strong stream of water, for three minutes after all the control (non-invasive) strain of yeast was completely washed away. If grown in the presence of sub-lethal rapamycin concentrations, washing was done after 5 days of growth (due to slower growth). In the second, more extensive wash method (also shown in Figure S4), plates were washed under a strong stream of water, with extensive rubbing with a gloved finger till even weakly invasive cells were completely removed. Pseudohyphal growth was observed after diploid yeast were allowed to grow for 2 weeks on SLAD medium agar plates by imaging under a 4× or 10× lens using a Nikon Eclipse 90i microscope.

### Purification of the Kog1/TORC1 complex and identification of complex components

Yeast cells expressing *KOG1*-flag were grown in the specified culture medium to either early log phase (in batch cultures) or to high density continuous chemostat cultures, collected by centrifugation, washed 1× in water and resuspended in lysis buffer containing 50 mM HEPES pH 7.5, 50 mM sodium fluoride, 10% glycerol, 150 mM potassium chloride, 0.5 mM EGTA, 0.5 mM EDTA, 2 mM sodium orthovanadate, 2 mM PMSF, 5 µM leupeptin, 2 µM pepstatin, 25× protease inhibitor cocktail solution (Roche), 0.5% Tween 20 (∼1 ml lysis buffer/50 OD_600_ units of cells). Typically, for larger scale purifications (with samples being visualized on gels stained with Coomassie blue or Silver stain) ∼150 (Silver stain) – 200 (Coomassie blue) OD_600_ units of cells were used. For smaller scale purifications used in co-immunoprecipitation experiments, ∼30–40 OD_600_ units of cells were used. Cells were lysed rapidly using a mini-beadbeater, by beating 5× for 20 second durations, with 30 second cooling incubations in between lysis cycles in an ice-water bath. Particular care was taken not to allow samples to warm up. Fresh PMSF (1 mM) was added immediately after lysis. Cells were centrifuged at 15000 g/4°C for 5 minutes, the supernatant was collected, re-centrifuged at the same speed for 5 minutes, and the supernatant collected. For purification, flag antibody was conjugated to magnetic protein G beads (Dynabeads, Invitrogen Corp.) in the same lysis buffer. Protein extracts were incubated with beads for not more than 45 minutes with gentle rocking at 4°C. Longer incubations resulted in a loss of proteins transiently interacting with the TORC1. A strong magnet was then used to attract the beads down, and the beads were washed 5× with 50 mM HEPES pH 7.5, 50 mM NaF, 10% glycerol, 150 mM KCl, 200 mM NaCl, 0.5 mM EGTA, 0.5 mM EDTA, 0.5% Tween20 and 1 mM PMSF. Washes were short (1–2 minute), longer washes resulted in a loss of complex components. However, multiple washes were performed to ensure specific interactions, absent in all control immunoprecipitates. For control immunoprecipitates detecting non-specific interactions, ∼15–20% more cells (compared to *KOG1*-flag samples) were lysed and prepared identically, in order to ensure that the identified TORC1-associated proteins were not advantageous interactions with the beads/IgG. Immunoprecipitations were carried out with the same magnetic bead-flag antibody conjugates. All residual wash buffer was removed, and the beads were boiled in reducing sample buffer before resolution on a 4–12% Bis-Tris SDS gel. Proteins were detected on the gel by standard colloidal Coomassie blue staining (SimplyBlue, Invitrogen) or silver staining (Invitrogen). Typical large scale purifications yielded ∼0.1–0.5 µg of Kog1/TORC1 (estimated approximately using BSA standards, not shown). Specific associated proteins were identified by excising the band from Coomassie stained SDS gels, digesting in-gel with trypsin, and subjecting the sample to nano HPLC/MS/MS on a ThermoFinnigan LTQ instrument, coupled with an Agilent 1100 Series HPLC system. The identified fragments were used to search against the yeast proteome database through the MASCOT search engine.

Co-immunoprecipitation experiments using HA and FLAG-tagged proteins were carried out using standard immunoprecipitation methods from yeast samples lysed and prepared as described above, followed by immunoprecipitation with one antibody and Western blot based detection of the second epitope. The primary antibodies used for epitope detection were anti-HA (Roche, #12CA5) and anti-Flag (Stratagene, #200472).

### Quantitative real-time PCR

For *FLO11* gene expression measurements in haploid yeast, the respective strains of haploid yeast were grown in YPD to an OD of ∼0.5, the cells were spun down and washed 2× in water, and subsequently processed for RNA extraction. For *FLO11* gene induction experiments under nitrogen starvation conditions, the respective diploid yeast wt or mutant strain was grown in SD to an OD_600_∼0.5, following which the cells were spun down and washed 2× in water, and transferred to synthetic medium containing no amino acids or nitrogen source other than 40 µM ammonium sulfate, along with 1% dextrose. The cells were grown under these conditions overnight after which they were harvested and processed for RNA extraction. Total RNA was isolated from these cells using a Masterpure yeast RNA extraction kit (Epicentre biotechnologies), treated with DNAse I, and the quality of RNA was verified. Subsequently, cDNA synthesis was carried out using 4 µg RNA and SuperScript II reverse transcriptase (Life Technologies). Ten ng of cDNA was used as a template for real-time quantitative PCR. The primers were designed using the Primer3 program, and the following primers were used: *FLO11-1* 5′- GTTCAACCAGTCCAAGCGAAA-3′, *FLO11-2* 5′- GTAGTTACAGGTGTGGTAGGTGAAGTG-3′, *ACT1-1* 5′-TCGTTCCAATTTACGCTGGTT-3′, *ACT1-2* 5′-CGGCCAAATCGATTCTCAA- 3′. The *FLO11* gene expression level was correlated to the reference gene *ACT1*. 10 µl RR-qPCR reactions were performed in a 7900HT Fast Real-Time PCR system (Applied Biosystems) using the Power SYBR Green reaction mix (Applied Biosystems). Specificity of amplicons was confirmed by generating melt curve profiles of all amplified products, and subsequent relative *FLO11*/*ACT1* level measurements were calculated and plotted using the GraphPad program using standard methods. The data presented were obtained from three independent samples/cultures for all strains and conditions presented.

## Supporting Information

Figure S1 Growth of wild type or *mks1*Δ, *kap123*Δ, *hef3*Δ, *ksp1*Δ and *uba4*Δ mutant strains of *S. cerevisiae* (Σ1278b background) on SD (yeast nitrogen base without amino acids+2% dextrose) media in the presence of 40 ng/ml rapamycin (measured after 48 hours).
(TIF)

Figure S2 Increased rapamycin resistance of *mks1*Δ mutant strains (CEN.PK background) that have been continuously maintained on YPD plates for several weeks (compare to Figure 2).
(TIF)

Figure S3 Nitrogen-independent invasive growth of haploid wild type (WT) or mutant Σ1278b background yeast strains under different growth conditions (also see Figure 4 and Figure S4). WT and mutant strains were streaked out on (A) Yeast extract/peptone/2% dextrose plates containing 1% Butanol, in the presence or absence of 40 ng/ml rapamycin or (B) yeast extract/peptone/2% dextrose plates with 0.1 mM 8-pCPT-2′O-Me-cAMPS (with or without 40 ng/ml rapamycin) or (C) yeast extract/peptone and 2% ethanol as the sole carbon source. Invasive growth was measured by washing plates under a steady stream of water after 72 hours of growth. Plates were washed continuously for 3 minutes after all non-invasive yeast streaks (CEN.PK genetic background) was washed away. *uba4*Δ strains do not show invasive growth in the presence of 1% butanol, or when grown on YPD/YPD+cAMP, but appear to show some invasive growth when grown on 2% ethanol. *kap123*Δ strains appear show slightly diminished invasive growth when grown on YPD+butanol or YPD+cAMP. All other strains show substantial invasive growth under all invasive growth conditions tested. (D) WT Σ1278b background strains were hypersensitive to normally sub-lethal concentrations (40 ng/ml) of rapamycin when grown on yeast extract/peptone media with ethanol as the sole carbon source, and showed extremely poor growth under these conditions (insufficient to cause invasion).
(TIF)

Figure S4 Nitrogen-independent invasive growth of haploid wild type (WT) or mutant Σ1278b background yeast strains under different growth conditions (also see Figure 4 and Figure S3), observed after more extensive and stringent washing. WT and mutant strains were streaked out on (A) Yeast extract/peptone/2% dextrose plates containing 1% butanol, in the presence or absence of 40 ng/ml rapamycin or (B) yeast extract/peptone/2% dextrose plates with 0.1 mM 8-pCPT-2′O-Me-cAMPS (with our without 40 ng/ml rapamycin) or (C) yeast extract/peptone and 2% ethanol as the sole carbon source. Invasive growth was measured by washing plates under a steady stream of water along with extensive rubbing with a gloved finger after 72 hours of growth to remove even cells that had weakly invaded the agar. *uba4*Δ strains do not show invasive growth in the presence of 1% butanol, or when grown on YPD/YPD+cAMP, and can also be rubbed off the plates when grown on 2% ethanol. *kap123*Δ strains appear show slightly diminished invasive growth when grown on YPD+butanol or YPD+cAMP. All other strains show substantial invasive growth under all invasive growth conditions tested.
(TIF)

Figure S5 Nitrogen starvation-dependent diploid pseudohyphal growth in wild type (WT) and *mks1*Δ, *kap123*Δ, *hef3*Δ, *ksp1*Δ and *uba4*Δ mutant yeast strains from a Σ1278b genetic background. Two panels are shown for each strain, and the left panel shows 4× magnifications of a broad field of colonies, and the right panel shows a 10× magnification (with 1.5× digital magnification) of one or two colonies with more extensive pseudohyphal development seen in that specific strain. Compare to CEN.PK background strains (Figure 5).
(TIF)
