# Supplementary figures and images for: Multiple TORC1-Associated Proteins Regulate Nitrogen Starvation-Dependent Cellular Differentiation in Saccharomyces cerevisiae
Source: PLoS One. 2011 Oct 17;6(10):e26081. doi: 10.1371/journal.pone.0026081 (PMC3197150; doi:10.1371/journal.pone.0026081)

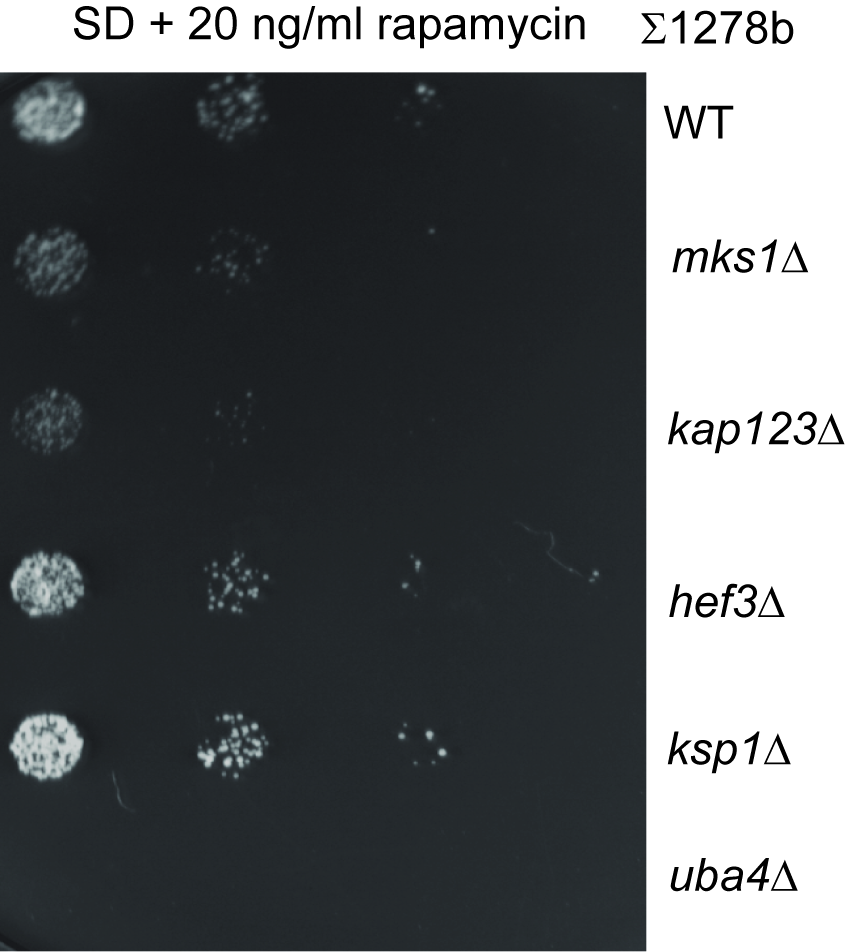

Supplement: Figure S1 — Growth of wild type or mks1Δ, kap123Δ, hef3Δ, ksp1Δ and uba4Δ mutant strains of S. cerevisiae (Σ1278b background) on SD (yeast nitrogen base without amino acids+2% dextrose) media in the presence of 40 ng/ml rapamycin (measured after 48 hours). (TIF) [file pone.0026081.s001.tif]

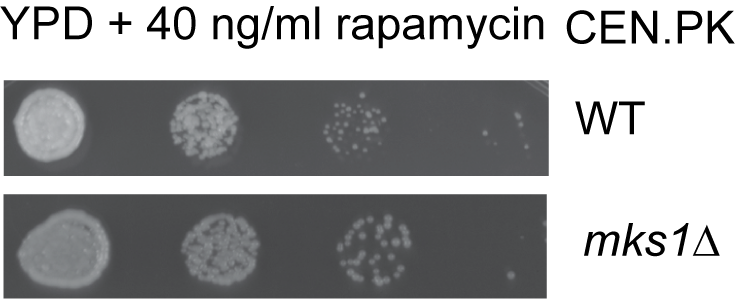

Supplement: Figure S2 — Increased rapamycin resistance of mks1Δ mutant strains (CEN.PK background) that have been continuously maintained on YPD plates for several weeks (compare to Figure 2). (TIF) [file pone.0026081.s002.tif]

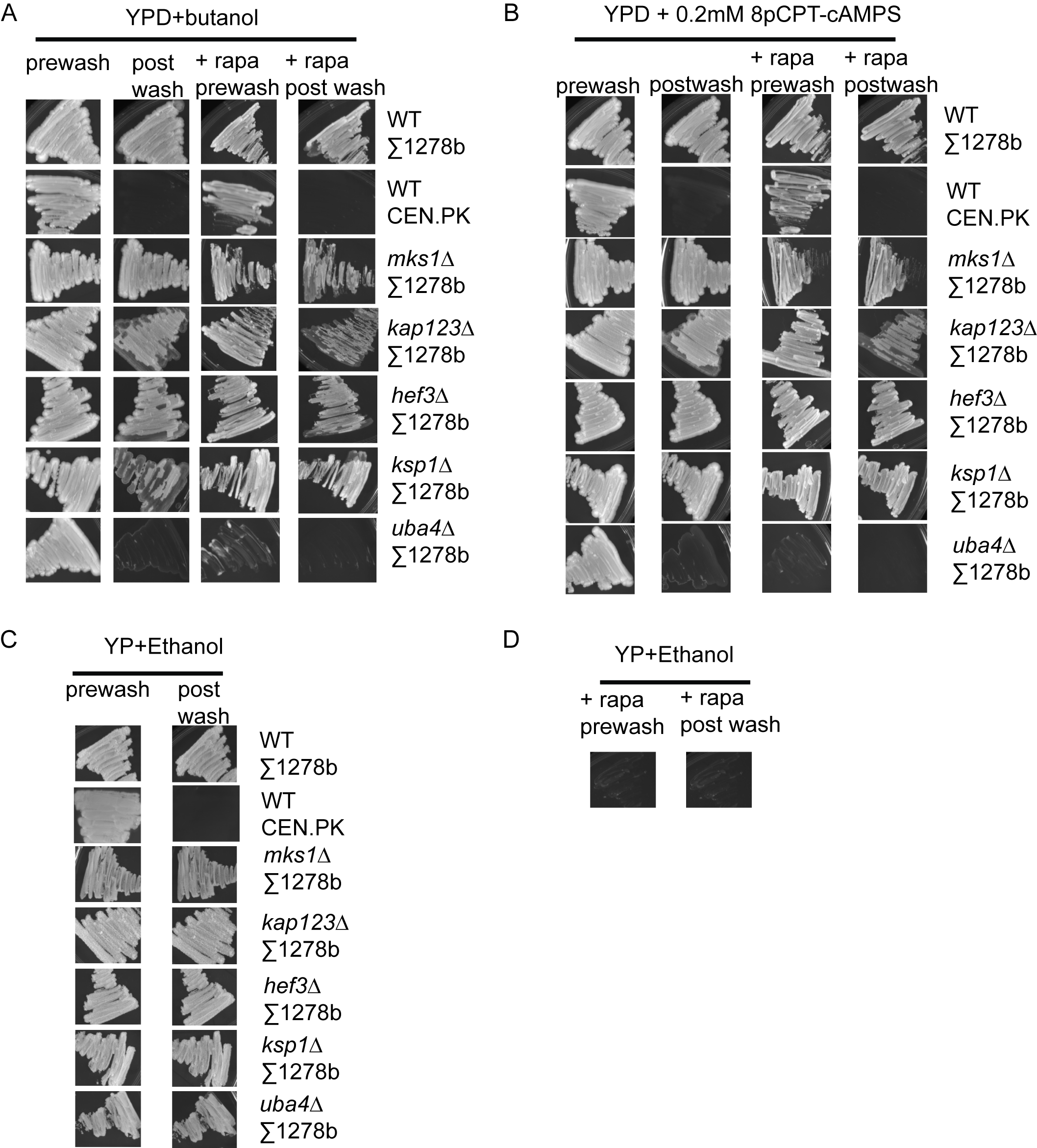

Supplement: Figure S3 — Nitrogen-independent invasive growth of haploid wild type (WT) or mutant Σ1278b background yeast strains under different growth conditions (also see Figure 4 and Figure S4). WT and mutant strains were streaked out on (A) Yeast extract/peptone/2% dextrose plates containing 1% Butanol, in the presence or absence of 40 ng/ml rapamycin or (B) yeast extract/peptone/2% dextrose plates with 0.1 mM 8-pCPT-2′O-Me-cAMPS (with or without 40 ng/ml rapamycin) or (C) yeast extract/peptone and 2% ethanol as the sole carbon source. Invasive growth was measured by washing plates under a steady stream of water after 72 hours of growth. Plates were washed continuously for 3 minutes after all non-invasive yeast streaks (CEN.PK genetic background) was washed away. uba4Δ strains do not show invasive growth in the presence of 1% butanol, or when grown on YPD/YPD+cAMP, but appear to show some invasive growth when grown on 2% ethanol. kap123Δ strains appear show slightly diminished invasive growth when grown on YPD+butanol or YPD+cAMP. All other strains show substantial invasive growth under all invasive growth conditions tested. (D) WT Σ1278b background strains were hypersensitive to normally sub-lethal concentrations (40 ng/ml) of rapamycin when grown on yeast extract/peptone media with ethanol as the sole carbon source, and showed extremely poor growth under these conditions (insufficient to cause invasion). (TIF) [file pone.0026081.s003.tif]

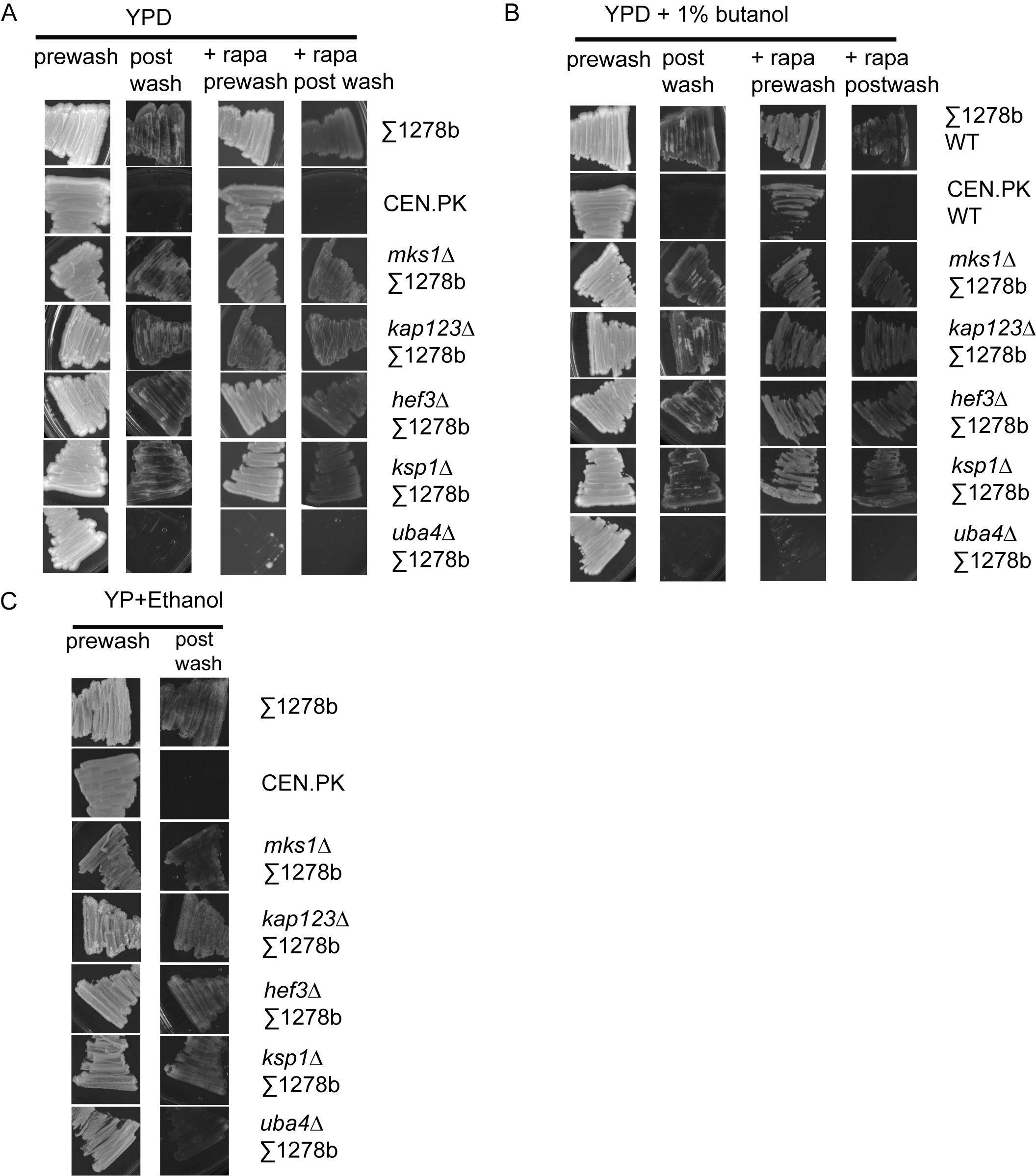

Supplement: Figure S4 — Nitrogen-independent invasive growth of haploid wild type (WT) or mutant Σ1278b background yeast strains under different growth conditions (also see Figure 4 and Figure S3), observed after more extensive and stringent washing. WT and mutant strains were streaked out on (A) Yeast extract/peptone/2% dextrose plates containing 1% butanol, in the presence or absence of 40 ng/ml rapamycin or (B) yeast extract/peptone/2% dextrose plates with 0.1 mM 8-pCPT-2′O-Me-cAMPS (with our without 40 ng/ml rapamycin) or (C) yeast extract/peptone and 2% ethanol as the sole carbon source. Invasive growth was measured by washing plates under a steady stream of water along with extensive rubbing with a gloved finger after 72 hours of growth to remove even cells that had weakly invaded the agar. uba4Δ strains do not show invasive growth in the presence of 1% butanol, or when grown on YPD/YPD+cAMP, and can also be rubbed off the plates when grown on 2% ethanol. kap123Δ strains appear show slightly diminished invasive growth when grown on YPD+butanol or YPD+cAMP. All other strains show substantial invasive growth under all invasive growth conditions tested. (TIF) [file pone.0026081.s004.tif]

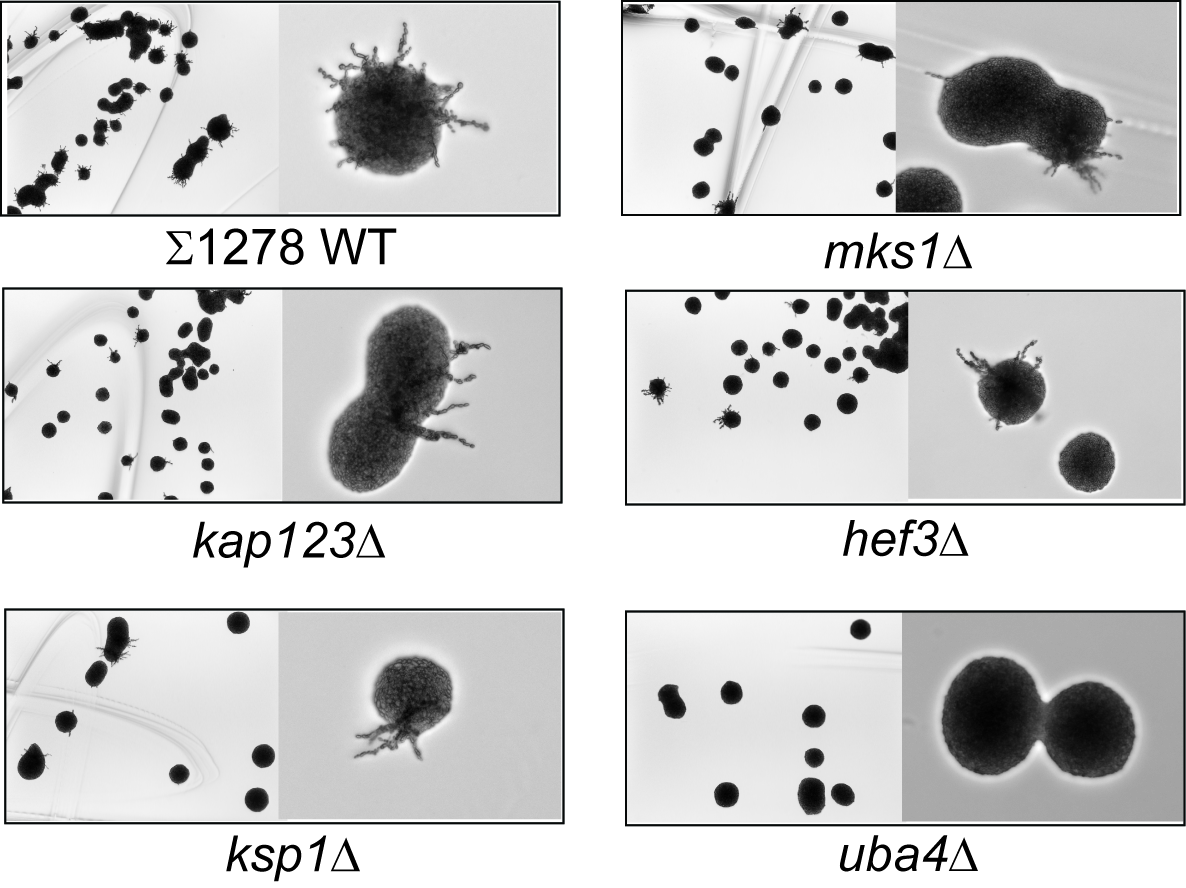

Supplement: Figure S5 — Nitrogen starvation-dependent diploid pseudohyphal growth in wild type (WT) and mks1Δ, kap123Δ, hef3Δ, ksp1Δ and uba4Δ mutant yeast strains from a Σ1278b genetic background. Two panels are shown for each strain, and the left panel shows 4× magnifications of a broad field of colonies, and the right panel shows a 10× magnification (with 1.5× digital magnification) of one or two colonies with more extensive pseudohyphal development seen in that specific strain. Compare to CEN.PK background strains (Figure 5). (TIF) [file pone.0026081.s005.tif]
